# Screening of MSI detection loci and their heterogeneity in East Asian colorectal cancer patients

**DOI:** 10.1002/cam4.2111

**Published:** 2019-04-03

**Authors:** Wenqi Bai, Jinfeng Ma, Yangyang Liu, Jing Liang, Yueqin Wu, Xuanqin Yang, Enwei Xu, Yan Li, Yanfeng Xi

**Affiliations:** ^1^ Department of Colorectal Surgery Shanxi Cancer Hospital Taiyuan, Shanxi P.R. China; ^2^ Department of General Surgery Shanxi Cancer Hospital Taiyuan, Shanxi P.R. China; ^3^ Department of Oncology Union hospital, Tongji Medical College, Huazhong University of Science & Technology Wuhan P.R. China; ^4^ Graduate School of Shanxi Medical University Taiyuan, Shanxi P.R. China; ^5^ Department of Pathology Shanxi Cancer Hospital Taiyuan, Shanxi P.R. China; ^6^ Department of Pathology Union hospital, Tongji Medical College, Huazhong University of Science & Technology Wuhan P.R. China

**Keywords:** colorectal cancer, intratumoral heterogeneity, microsatellite instability

## Abstract

**Objective:**

This study aims to screen the MSI detection loci suitable for the East Asian colorectal cancer patients. and explore its intratumoral heterogeneity.

**Methods:**

A total of 271 pathological tissues specimens of colorectal cancer were collected. The MSI status was detected using different PCR reagent kits with different detection loci. Then, the results were compared with the immunohistochemical (IHC) staining results. Microdissection of pathological tissues specimens detected to be MSI‐H was performed to examine whether there was intratumoral heterogeneity of MSI status.

**Results:**

Thirty‐nine out of 271 cases were dMMR. dMMR occurred mostly in patients with right‐hemi colon cancer (*P* < 0.0001). Compared with dMMR patients, the clinical stages of pMMR patients were more inclined to be in the late stage with lymph node metastasis (*P* < 0.0001). MSI‐H tumors were significantly associated with KRAS mutation (*P* = 0.036) and PD‐L1 expression (*P* = 0.038). Compared with Promega panel and 24‐locus detection, the consistency between NCI MSI panel and IHC staining results were the highest with the Kappa value of 0.850. The sensitivity of detection decreased from 87.18% to 56.41% with the increase in detection loci. Single locus analysis showed that the first two loci with the highest sensitivity were both mononucleotide loci, namely, BAT‐26 (95.45%) and BAT‐25 (86.36%). The dinucleotide locus with highest sensitivity was D2S123 (50%). The main detection loci of MSI‐H showed no intratumoral heterogeneity.

**Conclusion:**

The combination of 2 mononucleotide loci (BAT25, BAT26) and 3 dinucleotide loci (D2S123, D5S346, D17S250) might be the most suitable loci for MSI detection in East Asian population. There is no intratumoral heterogeneity in the main MSI loci.

## 1 INTRODUCTION

There may be significant differences in the genetic landscape, the response to chemotherapy and immunotherapy, as well as the classification and staging of tumors due to different status of microsatellite instability (MSI). Clarifying MSI status has an essential value in the diagnosis, treatment, and prognosis of colorectal cancer (CRC) and other tumors.^1^, ^2^, ^3^ MSI is generally resulted from the functional deficiency of one or more mismatch repair (MMR) proteins. On the contrary, microsatellite stability (MSS) was present when MMR is proficient (pMMR).^4^

The amount of microsatellites is huge in the human genome with over 19 million known loci,^5^ and are thus difficult to completely detect. Therefore, it is critical to screen a small amount of loci with high sensitivity and certain specificity for application in clinical practice. Currently, MSI detection mainly includes 2 major groups of loci, 1 consisting of 2 mononucleotide loci and 3 dinucleotide loci, and the other composed of 5 mononucleotide loci. MSI‐H (high) is defined as instability of 2 or more loci, MSI‐L (low) as instability of only 1 locus, and MSS no locus instability.^6^, ^7^ In addition, there are some other loci that have been used in the prior study,^8^ where the criteria for classification were as follows: (a) MSI‐H: >30% loci with instability, (b) MSI‐L: 10%‐30% loci with instability, and (c) MSS: less than 10% loci with instability.^9^ Despite its low sensitivity to fluorouracil, MSI‐H cancer was reported to have a much better response toward checkpoint inhibitors than MSI‐L or MSS cancer. Besides, MSI‐H plays an impotant role in the pathogenesis of Lynch syndrome, also known as hereditary nonpolyposis colorectal cancer (HNPCC). HNPCC is a familial hereditary disease caused by germline mutations in any 1 of 5 DNA MMR genes—*MLH1*, *MSH2*, *MSH6, *and *PMS2* and, rarely, *PMS1*, which result in MSI.^10^ This syndrome is associated with an increased risk of a great diversity of cancers, such as colorectal, endometrial, gastric, ovarian, small bowel, hepatobiliary as well as urothelial cancers, which may occur synchronously or metachronously. Identiying HNPCC patients is critical to perform firm cancer surveillance in these patients or their relatives. The detection of MSI status is thus of great significance.^11^ However, the screening of the 2 major groups of MSI loci is predominantly based on Caucasian population. Whereas, the incidences of MSI are quite different among different populations.^9^ The detection loci used by many researches in Asia are different, resulting in different detection rates of MSI.^12^, ^13^ Given the existence of racial differences, study screening the optimal loci for East Asian population is lacking. In this study, the sensitivities of multiple MSI detection loci in Chinese population were compared, followed by the consistency analysis of multiple loci combinations with immunohistochemical (IHC) staining results to screen the optimal detection loci for Chinese.

Intratumoral heterogeneity and clonal evolution of tumors have attracted general attention in recent decades. Several studies have confirmed that there is genomic heterogeneity in different sites of 1 tumor mass, as well as between primary and metastatic foci.^14^, ^15^, ^16^ The presence of intratumoral heterogeneity is suggested to be associated with drug resistance and treatment failure.^17^, ^18^ Therefore, it is generally recommended that multiple biopsies and re‐biopsy after treatment failure should be conducted to build an objective and effective therapeutic regimen. Whether there is intratumoral heterogeneity of MSI status has not yet been reported in the existed studies. This study preliminarily explored the intratumoral heterogeneity of MSI, and analyzed the relationship between MSI status and driver genes of CRC (*KRAS, NRAS, BRAF*). In addition, the programmed cell death protein 1 ligand (PD‐L1) is an important factor in tumor microenvironment and is associated with the therapeutic effect of immunotherapy.^19^ The correlation between PD‐L1 and MSI status was also analyzed in this study.

## 2 MATERIALS AND METHODS

### 2.1 Human samples

This was a multi‐centered retrospective study which incorporated 271 patients with CRC from Shanxi Cancer Hospital, Changhai Hospital of Shanghai, and Wuhan Union Hospital, from January 2017 to January 2018. Basic and clinicopathological information from all patients were included, while mutation detection was performed in 200 out of 271 patients. The participants were screened with the following criteria: sporadic CRC diagnosed by pathology; acceptance of radical or local surgical treatment; and availability of detailed clinical and pathological data for statistical analysis, including the degree of differentiation, mucinous differentiation, infiltration of lymphocytes, growth patterns, lesion locations, and other indicators. We collected paraffin‐embedded sections from the selected patients, including tumor tissue and paired normal tissue sections (5 μm thick). All samples were routinely formalin fixed and paraffin embedded as follows. First, fresh tumor tissues were fixed with 10% neutral buffered formalin (10%formalin, 29 mmol/L NaH_2_PO_4_, 46mmol/L Na_2_HPO_4_) for 6‐48 hours. Then, dehydration procedures were performed with tissues in 10% neutral buffered formalin for 1 hour, in series of 75%‐100% ethanol solutions for a total of 6 hours, in xylene for 45 minutes twice, and in paraffin for 1 hour three times. Those dehydrated tissues were then taken out, and put into the melted paraffin (70°C) and finally froze at −5°C to solidify the samples. All of the enrolled patients (100%) were Asians.

This study was approved by the Ethics Committee of Shanxi Cancer Hospital, and the study was in compliance with the Declaration of Helsinki Principles.

### 2.2 Immunohistochemical detection

The expression of 4 mismatch repair proteins MLH1, MSH2, MSH6, and PMS2, were detected in all 271 tissue specimens. Sections with a thickness of 3‐5μm were cut and mounted on glass slides. Loaded slides were dried in 70°C for one hour. Deparaffinization was then carried out in xylene and rehydration in a graded ethanol series, followed by antigen retrieval steps. Samples were boiled in ethylenediamine tetra‐acetic acid (EDTA) solution (pH 9.0) in a pressure cooker for 10 minutes. The slides were then cooled to room temperature (RT), washed using deionized water and soaked in phosphate buffered saline (PBS). Slides were incubated in a 4°C thermostat overnight after treated with primary antibodies (MLH1 mouse monoclonal antibody (MAb)：ES05, MAB‐0789; MSH2 rabbit MAb: RED2, RMA‐0776; MSH6 rabbit MAb: EP49, RMA‐0770; PMS2 rabbit MAb: EP51, RMA‐0775; MXB® Fuzhou, China). The slides were then exposed in RT for 45 minutes and washed by PBS. Samples were incubated with secondary MAb‐DAKO REALTM EnVisionTM HRP RABBIT/MOUSE (ENV)‐( K5007, DAKO) in RT for 1 hour. DAB (3,3N‐Diaminobenzidine Tetrahydrochloride) color development kit (DAB‐1031, MXB® Fuzhou, China) was used for immunoreaction (10 minutes) visualization following the manufacturer's protocols. After being washed in flowing tap‐water for 3 minutes, all sections were stained with hematoxylin for 1 minute and then washed in flowing tap‐water for another 3 minutes. Slides were immersed in 1% hydrochloride ethanol solution for 5 seconds, washed in flowing tap‐water for 3 minutes, immersed in saturated lithium carbonate solution for 1 minute, washed in flowing tap‐water for 10 minutes, dehydrated in a graded ethanol series, and then immersed in xylene. Finally, those slides were sealed by neutral gum. Slides were observed under the microscope (400×, CX31, OLYMPUS, Japan) and signals were classified as negative or positive. In addition, according to the deletion of different proteins,^9^ tissue samples were categorized into 2 types, that is, dMMR and pMMR. Figure S1 showed the pathological sections with negative protein staining as well as positive staining for each protein, respectively.

Mouse anti‐human monoclonal antibody Dako 22C3 was applied as a primary antibody to recognize PD‐L1. PD‐L1 was mainly expressed in the cytoplasm in both tumor cells and lymphocytes, and PD‐L1 positive was recorded when there was staining in the above‐mentioned areas.

### 2.3 MSI detection

MSI detection was performed in all of the 271 specimens with 21 loci simultaneously, which included 5 loci in NCI MSI panel and other 16 loci. DNA extraction was performed using a formalin‐fixed paraffin‐embedded whole genome extraction kit (cat. no. 180134, Qiagen, Hilden, Germany). The NCI MSI panel included 2 mononucleotide repeat markers, 3 dinucleotide repeat markers, and 1 pentanucleotide repeat marker (Penta C). Mononucleotide and dinucleotide markers are used for MSI determination, and the pentanucleotide marker is used to detect potential sample mixups or contamination. The detection loci of NCI MSI panel are listed in Table 1. Those other 16 loci included: *BAT‐40, AFM119xh12a, 52H10, AMF183yc3 (D3S1283), 50C10, Mfd28CA (D10S89), AMF249xbla (D13S175), Mfd41 (D17S261), Mfd26CA (D18S34), AMF08lza5 (D11S904), AMF248yf1 (D18S69), AMF218xela (D11S1318), AMF164xe31a (D18S58), AMF.164xe31a (D9S171), D1, TP53.PCR15.1*. The chromosomal locations, primer sequences and products’ lengths of those 16 loci were detailed in Table S1.

**Table 1:** Detection loci of NCI MSI panel

NCI MSI panel loci (n = 5)	
Mononucleotide (n = 2)	Dinucleotide (n = 3)
*BAT‐25*	*D2S123*
*BAT‐26*	*D5S346*
	*D17S250*

PCR amplification was performed using a 10 μL reaction volume, including 2 × 5 μL PCR Master Mix, 5 × 2 μL 5 primer mix, 0.2 μL Amplitaq Gold DNA polymerase (5 units/μL), and 5‐10 ng DNA templates. PCR was performed on a PE 9600 thermal cycler using the following cycling profiles: 95°C holds for 4 min, 30 cycles at 95°C for 30 s, 60°C for 30 s, 72°C for 30 s, and 60°C for 45 min, then hold. Holding for 30 cycles avoided the formation of shadow peaks. After amplification, the PCR products were detected and analyzed using an ABI 3730 genetic Analyzer (Applied Biosystems, CA) following the manufacturer's protocols. Data were analyzed furtherly with GeneScan Analysis and Genotyper Software packages from Applied Biosystems to identify the predominant allele size for each locus. MSI positivity was determined by the number of allelic bases within corresponding loci, and by the internal control index of the tumor samples compared to their paired normal control samples.

### 2.4 Assessing of MSI using the panel of 5 mononucleotide repeats loci

Among the 271 specimens, the detection of MSI with Promega panel (MSI Analysis System, Version 1.2, Promega Corporation, WI) was conducted in 72 samples, which included 5 mononucleotide repeat markers (*BAT‐25, BAT‐26, NR‐21, NR‐24* and *MONO‐27*). The detection was carried out according to the manufacturer's instructions.

### 2.5 BRAF, KRAS, NRAS mutation detection

Real‐time quantitative fluorescence PCR was performed to detect Kinase‐related mutations. *KRAS* and *NRAS* detection kit (ADx‐KN03‐MX, AmoyDx, Xiamen, China) was used to detect the mutations on exon 2, 3, 4 of *KRAS* and *NRAS* *BRAF* detection kit (ADx‐BR01, ADx‐BR02, AmoyDx, Xiamen, China) was used to detect mutation V600E on exon 15 of *BRAF*. The PCR amplification procedures were as follows:

**Table:** 

Step	Temperature	Time	Cycle
1	37°C	10 min	1
2	95°C	5 min	1
3	95°C	15 s	40
	60°C	60 s（collecting 6‐FAM and HEX signals）	

The above PCR processes were performed at Fluorescent Quantitative PCR instrument of ABI 7500. 6‐FAM (6‐carboxy‐fluorescein) and HEX (5‐hexachloro‐fluorescein) signals were collected and results were interpreted according to manufacturer's protocals. In brief, for *KRAS* and *NRAS* detection, ΔCt (cycle threthold) ≤10 and ΔCt ≤ 12 were recorded as positive, while ΔCt ＞14 was defined as negative. For *BRAF* V600E detection, Ct ＜28 was defined as positive, whereas Ct ≥28 was defined as negative with a internal control ranging from 10 to 20.

### 2.6 MSI intratumoral heterogeneity detection

Pathological sections were stained with hematoxylin‐eosin (HE). Tumor tissues were divided into different regions by connective tissues when observed under the microscope. Delineate and then mark each divided region. The thickness, HE staining, and microscopic observation procedures were the same with that described in IHC detection part. Referring to the delineated slides, tumor tissues were scraped from the corresponding regions of the unstained pathological slides. DNA was then extracted, and MSI status detection was carried out following the procedures described in MSI detection part.

### 2.7 Statistical analysis

In the comparison between groups of pMMR with dMMR as well as MSI‐L/MSS with MSI‐H, metric data were analyzed by Mann‐Whitney U test, and categorical data were compared using chi‐square test (*T* ≥ 1) or Fisher exact probability test (*T *< 1). Kappa consistency test was used to analyze the consistency of MSI detection results with different combinations of loci being studied. Statistical analysis was carried out using SPSS22.0 (IBM Corp., Somers, NY) software. *P* values were two‐tailed with a value of ＜0.05 as statistically significant.

## 3 RESULTS

### 3.1 Baseline characteristics

The basic and clinicopathological information of the enrolled patients was shown in Table 2. The median age of 271 patients was 58 years old, and the earliest onset was 18 years old. There were 147 (54.24%) middle‐aged and young patients (under 60 years old). The incidence of dMMR was 14.39%. As for CRC location, rectum was the most frequent site, followed by the right‐hemi colon, and then left‐hemi colon. Tumor grades of 173 cases were known, of which grade Ⅱ had the highest proportion (84.83%). Ninety‐nine cases had lymph node metastasis and 8 cases had distant or implantation metastases. There were 200 cases with available *RAS* mutation information, of which 161 patients were pMMR or MSI‐L/MSS and 39 patients were dMMR or MSI‐H. The percentage of *BRAF* mutation was 4.5% with the same variation type of p.V600E, and *KRAS* and *NRAS* mutation rates were 26% and 1.5%, respectively. In addition, *BRAF* mutation was exclusively detected in patients with *KRAS* or *NRAS* mutation.

**Table 2:** Relationship of MMR and MSI status with clinicopathological features of patients

Items	Total (n = 271) (%)	pMMR (n = 232) (%)	dMMR (n = 39) (%)	*P* value	MSI‐L/MSS (n = 232) (%)	MSI‐H (n = 39) (%)	*P* value
Gender (Female)	111 (40.96)	91 (39.22)	20 (51.28)	0.157	93 (40.09)	18 (46.15)	0.476
Age (Range/median)	18‐84/58 y	18‐83/61yr	25‐84/61 y	0.573	18‐84/61 y	25‐82/61 y	0.570
Tumor location							
Right‐hemi colon	68 (25.09)	49 (21.12)	19 (48.72)	<0.001	47 (20.26)	21 (53.85)	<0.001
Left‐hemi colon	66 (24.35)	60 (25.86)	6 (15.38)	0.158	60 (25.86)	6 (15.38)	0.158
Transverse colon	9 (3.32)	8 (3.45)	1 (2.56)	0.776	8 (3.45)	1 (2.56)	0.776
Multiple sites	1 (0.37)	1 (0.43)	0	0.681	1 (0.43)	0	0.681
Rectum	131 (48.34)	118 (50.86)	13 (33.33)	0.043	120 (51.72)	11 (28.21)	0.007
Grade							
Grade I	1 (0.56)	1 (0.62)	0	>0.999	1 (0.62)	0	>0.999
Grade II	151 (84.83)	140 (86.42)	11 (68.75)	0.060	143 (88.27)	8 (50)	<0.001
Grade II‐III	25 (14.04)	21 (12.96)	4 (25)	0.186	18 (11.11)	7 (43.75)	<0.001
Grade III	1 (0.56)	0	1 (6.25)	0.090	0	1 (6.25)	0.090
TNM stage							
Stage I	75 (27.68)	56 (24.14)	19 (48.72)	0.002	56 (24.14)	19 (48.72)	0.002
Stage II	95 (35.06)	78 (33.62)	17 (43.59)	0.227	78 (33.62)	17 (43.59)	0.227
Stage III	93 (34.32)	90 (38.79)	3 (7.69)	<0.001	90	3	<0.001
Stage IV	8 (2.95)	8 (3.45)	0	0.607	8 (3.45)	0	0.607
Mucinous adenocarcinoma	81 (29.89)	71 (30.60)	10 (25.64)	0.531	71 (30.60)	10 (25.64)	0.531
Lymph node metastasis	99 (36.53)	96 (41.38)	3 (7.69)	<0.001	96 (41.38)	3 (7.69)	<0.001
Distant/implantation metastasis	8 (2.95)	8 (3.45)	0	0.607	8 (3.45)	0	0.607
PD‐L1 positive	40 (14.76)	32 (13.79)	8 (20.51)	0.274	30 (12.93)	10 (25.64)	0.038
Mutation type (n = 200)							
*KRAS*	52 (26)	46 (28.57)	6 (15.38)	0.092	47 (29.19)	5 (12.82)	0.036
*NRAS*	3 (1.5)	3 (1.86)	0	>0.999	3 (1.86)	0	>0.999
*BRAF*	9 (4.5)	6 (3.73)	3 (7.69)	0.284	6 (3.73)	3 (7.69)	0.284

Note

The comparison of patients’ ages were analyzed by Mann‐Whitney *U* test. Categorical data were compared by using χ^2^ test(*T* ≥ 1) or Fisher exact probability test (*T *< 1).

### 3.2 Comparison between pMMR group and dMMR group

Eligible patients were divided into 2 groups as dMMR and pMMR according to IHC staining to identify their differences in clinicopathological characteristics. The characteristics of the 2 groups were compared and corresponding results were shown in Table 2. The results showed that the incidence of dMMR in right‐hemi colon cancer was significantly higher than that in other sites (*P* ＜ 0.001). Whereas, the incidence of pMMR in rectal cancer was higher than that in other parts (*P* = 0.043). Besides, dMMR had a slightly higher incidence in stage Ⅱ tumors. However, the results were not statistically different in this study. dMMR had a relatively high incidence in patients with stage Ⅰ CRC (*P* = 0.002), and slightly higher incidence in patients with stage Ⅱ CRC, yet without statistical difference. Compared to patients with dMMR, the incidence of advanced CRC was significantly lower in pMMR group. Besides, lymph node metastasis was more frequently seen in patients with pMMR (*P* ＜ 0.001).

### 3.3 Comparison between MSI‐H group and MSI‐L/MSS group

In order to clarify the difference of clinical and pathological characteristics due to different MSI statuses, enrolled patients were divided into 2 groups as MSI‐H and MSI‐L/MSS based on the detection results of NCI MSI panel. The data of these 2 groups were compared and shown in Table 2. Corresponding results indicated that MSI‐H occurred more commonly in tumors at the right‐hemi colon (*P* ＜ 0.001), while MSI‐L/MSS in the rectal cancer (*P* = 0.007). MSI‐H was majorly seen in grade Ⅱ and stage Ⅰ cancer, whereas MSI‐L/MSS was majorly in grade Ⅱ‐Ⅲ and stage Ⅲ‐Ⅳ cancer. Meanwhile, MSI‐H tumors generally had lymph node metastasis (*P* ＜ 0.001). A much higher PD‐L1 positive rate was seen in MSI‐H tumors (*P* = 0.007). However, there was no significant difference of *BRAF* mutation incidences between the 2 groups. The frequency of *KRAS* mutation was higher in MSI‐L/MSS group (*P* = 0.036).

### 3.4 Consistency analysis

Based on the currently recognized classification, MSI‐L and MSS are classified as 1 type corresponding to pMMR, and MSI‐H is classified as the other type corresponding to dMMR. As for determination of MSI‐H status testing more than 5 loci, there were 2 main broken points currently. More than 30% or over 20% of loci varied can be recorded as MSI‐H,^9^, ^20^ with the former one as the working standard. Figure S2 showed the representative figures of MSI detection results.

#### 3.4.1 Consistency analysis of NCI MSI panel and all 24‐locus detection

To determine whether there was statistical difference between multi‐locus detection and 5‐locus detection using NCI MSI panel, the results of all 24 loci were aggregated and Kappa consistency test was then conducted. As shown in Table 3, the Kappa value was 0.711 when 30% was taken as the MSI‐H broken point in the 24‐locus detection.

**Table 3:** Consistency analysis of NCI MSI panel and total 24‐locus detection

Consistency analysis		NCI MSI panel		Kappa value
		MSI‐L or MSS	MSI‐H	
Total 24‐locus detection (Break point: 30%)	MSI‐L or MSS	232	16	0.711
	MSI‐H	0	23	

Note

Kappa consistency test was used to analyze the consistency of different locus test results.

#### 3.4.2 Consistency analysis of NCI MSI panel, Promega panel (5‐locus mononucleotide), and all 24‐locus detection with IHC results

To illustrate the consistence of detection results by different panels and IHC results, the consistency analysis was carried out. As described in Table 4, the consistency was the best between NCI MSI panel detection and IHC result. Besides, the 24‐locus detection with 30% as break point exhibited the poorest consistency with IHC results.

**Table 4:** Consistency analysis of NCI MSI panel, Promega panel, and total 24‐locus detection with IHC as a reference

	pMMR	dMMR	Kappa value
NCI MSI panel			
MSI‐L or MSS	227	5	0.850
MSI‐H	5	34	
Promega panel (n = 72)			
MSS	52	4	0.769
MSI‐H	2	14	
Total 24‐locus detection (Break point: 30%)			
MSI‐L or MSS	231	17	0.675
MSI‐H	1	22	

Note

Kappa consistency test was used to analyze the consistency of different locus test results.

#### 3.4.3 Sensitivity and specificity analysis of NCI MSI panel, Promega panel, and all 24‐locus detection

Table 5 showed the sensitivities, specificities, positive predictive values, and negative predictive values of the 3 combinations of different detection loci. Generally, the detection results of NCI MSI panel were better than that of the other 2 loci combinations. In particular, the sensitivity of NCI MSI panel was obviously higher than that of the other 2 combinations.

**Table 5:** Sensitivities and specificities of NCI MSI panel, Promega panel, and total 24‐locus detection

	Sensitivity	Specificity	Positive predictive value	Negative predictive value
NCI MSI panel	87.18%	97.84%	87.18%	97.84%
Promega panel (n = 72)	77.78%	96.30%	87.5%	92.86%
Total 24‐locus detection (Break point: 30%)	56.41%	99.57%	95.65%	93.55%

### 3.5 The top 10 loci with highest sensitivity in 24 loci

**Figure 1:**
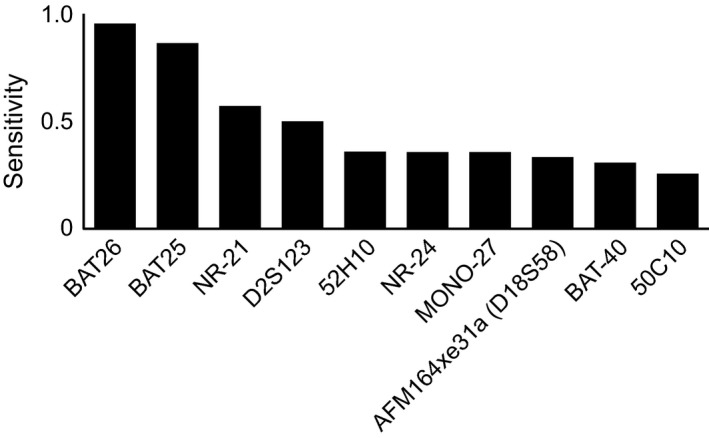
The 10 loci with the highest sensitivity in MSI detection. The top 10 loci with the highest sensitivity were counted in 24 loci so as to identify the sensitivity of each locus by MSI detection. The first 2 loci with the highest sensitivity were mononucleotide loci, namely, *BAT‐26 *and *BAT‐25*. The dinucleotide locus with the highest sensitivity was *D2S123*. These 3 loci were all contained in the detection of NCI MSI panel

The most sensitive 10 loci were listed out in Figure 1. The 2 loci with the highest sensitivity were *BAT‐26* and *BAT‐2*5, both of which were mononucleotide loci. The dinucleotide locus with the highest sensitivity was *D2S123*. These 3 loci were all contained in NCI MSI panel.

### 3.6 MSI intratumoral heterogeneity detection

**Figure 2:**
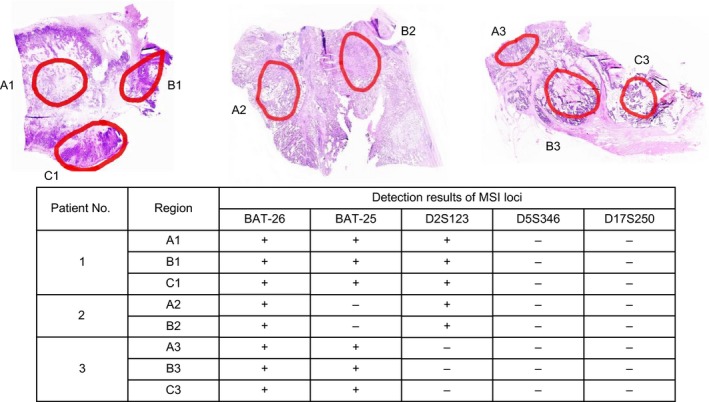
There was no heterogeneity of MSI in different regions of the tumor (+, positive; −, negative) (40 × 0.31). Pathological sections were stained with hematoxylin‐eosin (HE). Tumor tissues were divided into different regions by connective tissues when observed under the microscope. Delineate and then mark each divided region. The thickness, HE staining and microscopic observation procedures were the same with that described in IHC detection part. Referring to the delineated slides, tumor tissues were scraped from the corresponding regions of the unstained pathological slides. Then, the DNA was extracted from corresponding region. MSI status detection was carried out by PCR technology. A1, B1, and C1 were 3 different regions from a tumor slide. The results of MSI detection were all MSI‐H, besides, the mutation loci were *BAT26*, *BAT25,* and *D2S123*. A2 and B2 were 2 different regions of tumor tissues from another patient. The results of MSI detection were all MSI‐H, and the mutation loci were *BAT26* and *D2S123*. Additionally, A3, B3, and C3 were 3 different regions of tumor tissues from the third patient. The results of MSI detection showed MSI‐H, and the mutation loci were *BAT26* and *BAT25*

To clarify whether intratumoral heterogeneity of MSI‐H status exists, all MSI‐H samples were dissected by regions. NCI MSI panel was applied for MSI detection, and the results showed that there was no intratumoral heterogeneity of MSI‐H (Figure 2).

## 4 DISCUSSION

This study aims to screen the MSI detection loci suitable for the East Asian CRC patients, and to explore the intratumoral heterogeneity of MSI major detection loci. As described previously, the screening of 2 major groups of MSI loci is predominantly based on the study of Caucasian population. Corresponding data was lacking of Asians. To our knowledge, this is so far the largest study to screen MSI loci in Asian CRC patients. Besides, this is the first study to explore intratumoral heterogeneity of MSI major detection loci.

Detection of MSI status is of great importance in the diagnosis and treatment of CRC. Studies have shown that MSI‐H has a higher incidence in right‐hemi colon cancer and stage Ⅱ CRC compared with MSS or MSI‐L. Meanwhile, patients with MSI‐H have a better prognosis, accompanied by a less tendency to lymph node and distant metastasis, and a lower sensitivity to fluorouracil, but a higher sensitivity to irinotecan than that of MSS or MSI‐L patients. Besides, MSI‐H is closely related to Lynch syndrome.^9^ Similar to the results of other studies, this study showed that the incidence of MSI‐H in right‐hemi colon CRC was significantly higher than CRC in other parts. Besides, there was a lower rate of lymph node metastasis in MSI‐H CRC than in MSI‐L or MSS CRC. In this study, dMMR had a relatively high incidence in stage Ⅰ CRC while slightly higher in stage Ⅱ CRC, but no statistical difference was observed in the latter. In addition, the incidence of dMMR was significantly lower in advanced CRC than pMMR. What is more, the incidences of both dMMR and MSI‐H were statistically lower in rectal cancer in our study.

There is an obvious difference in the incidences of MSI‐H among different races. In the Caucasian population, the incidence of MSI‐H is about 8%‐20% in CRC, and even up to 45% among African Americans.^17^, ^21^, ^22^ However, in China, it is generally less than 10%.^23^, ^24^ In this study, the incidence of MSI‐H was 14.39%. Given the difference of MSI‐H rates among different populations, it is important to screen MSI loci suitable for detection in Chinese population. This is the first large‐scale screening study of MSI loci based on Chinese population, which showed that NCI MSI panel had the best consistency with the results of IHC.

An increase in the amount of detection loci has no obvious advantage in judging the status of MSI. In fact, it can impair the accuracy of MSI determination. In this study, the results of 24 detection loci showed that although its specificity was slightly higher than that of 5‐locus detection panels, its sensitivity decreased remarkably to 56.41%, with a poor consistency with the results of IHC. In addition, consistency analysis suggested that the false negative rate increased significantly as the number of detection loci increased to 24. Considering that genetic mutations due to MSI is a cumulative process and there are differences in the frequency, onset, and order of mutations. Thus, inclusion of excessive mutation loci occurring at late stage or with relatively low mutation frequencies may lead to a decrease in the sensitivity of MSI detection. Based on the results of this study, variations of *BAT‐25* and *BAT‐26* are speculated to be the most common and earliest events, which are universal in MSI‐H CRC applicable for detection. It is noteworthy that blindly increasing the amount of MSI detection loci may have a negative impact on the detection of MSI, which in turn underlines the great clinical value of loci screening.

There was a comprehensive analysis about the sensitivities and specificities of the 3 different combinations of detection loci in this study. Results indicated that the outcome of NCI MSI panel was optimal, and its sensitivity was obviously higher than that of the other 2 loci combinations. In addition, the sensitivity analysis of each single locus showed that the most sensitive loci were 2 mononucleotide loci, that is, *BAT‐25* and *BAT‐26*, and the most sensitive dinucleotide loci was “*D2S123*,” all 3 of which were included in the detection range of NCI MSI panel. Furthermore, this study indicated that the 5 MSI detection loci in NCI MSI panel are optimal for Chinese population.

In recent years, tumor heterogeneity and clonal evolutionhave become a hot topic. Sequencing analysis showed that there was intratumoral heterogeneity in the solid tumor genomics.^25^, ^26^ However, studies on intratumoral heterogeneity of the main detection loci of MSI were lacking. Mutations in *MLH1*, *MSH2*, *MSH6, PMS2,* and other MMR related genes, promoter methylation and epigenetic changes resulted in the loss of MMR protein, which eventually led to the presence of MSI‐H in CRC. This kind of genetic defect partly originated from germline variation and existed in all cells of the body, therefore, there was no intratumoral heterogeneity. Some others originated from somatic mutations, and may occur at the initial stage in carcinogenesis acting as driver factors, and no intratumoral heterogeneity existed consequently. This theory was also illustrated in the progression model of CRC described by Vilar E et al.^9^

Due to the existence of intratumoral heterogeneity, tumors are prone to develop drug resistance, leading to treatment failure. Since there is no intratumoral heterogeneity in the MSI‐H status, MSI‐H tumors respond to immunotherapy uniformly and stably, with a relatively low probability of drug resistance or treatment failure. In CheckMate‐142, for patients with metastatic CRC in MSI‐H status, the median response duration did not reach when Nivolumab was applied.^27^ In addition, when detecting driver genes of tumors, it is recommended to take multiple biopsies to avoid missing detection. However, it is unnecessary to detect multiple sites repeatedly for MSI determination since there is no intratumoral heterogeneity in its main detection loci. One‐time examination is representative and the test result has high reliability. There is no related study on whether MSI status will change during treatment. Hence, further tests are needed to clarify dynamic changes of MSI status during tumor treatment.

Combined detection of different biomarkers may contribute to more accurate prediction of treatment effects and prognosis for cancer patients. PD‐L1 is also a biological factor that can predict the efficacy of immune checkpoint inhibitors. However, some patients with low‐expression or negative PD‐L1 can also benefit from immune checkpoint inhibitors. It is clinically significant to filter these patients out. CheckMate‐142 study showed that patients with dMMR or MSI‐H, regardless of the expression of PD‐L1, can benefit from immune checkpoint inhibitors.^27^ Therefore, the detection of MSI status can make up for the detection of PD‐L1 in predicting the efficacy of immune checkpoint inhibitors. In addition, MSI‐H is more likely to appear in tumor tissues where PD‐L1 is expressed in tumor cells and interstitial immune cells within tumors. When there is no expression of PD‐L1 in tumor cells, there is no significant difference in MSI status whether or not it is expressed in interstitial immune cells.^28^ In this study, the expression of PD‐L1 in MSI‐H group was obviously higher than that in MSI‐L/MSS group, which further confirmed that MSI status was related to the expression of PD‐L1. MSI‐H was also associated with a good prognosis of CRC, but not all CRC patients with MSI‐H had good outcome.^29^ MSI‐H CRC patients with PD‐L1 expressed in tumor cells might be those with poor prognosis.^28^

In this study, the relationship between MSI status and kinase encoding gene mutations was also explored. In 200 specimens, the percentage of *BRAF* mutation (all p.V600E) was 4.5%, which was lower than that of other study (8.2%),^30^ with higher incidence in dMMR group and MSI‐H group. However, there was no statistical difference in this result due to the small sample size. In addition, the frequency of *KRAS* mutation was lower in MSI‐H group. It was considered that *KRAS* mutation was a key event in the early stage of carcinogenesis, therefore, it was mutually exclusive with dMMR or MSI‐H, which is also a key event in the early stage of carcinogenesis.^31^

This is so far the largest MSI loci screening and first intratumoral heterogeneity exploring study in Asian CRC patients. However, there were 3 limits in interpreting our results. First, the therapeutic information was perplexing and difficult to categorize. Therefore, we were unable to define the relationship between MSI status and treatment efficacy. Second, this was a retrospective study and follow‐up data were lacking. So, we could not compare clinical outcomes of patients with different MSI status. Third, the participants in this study were consecutively enrolled from 3 hospitals in different regions of China, and there may be a bias to apply the results to East Asian population.

In conclusion, this study focuses on screening of MSI loci in Chinese population. To sum up, the combination (NCI MSI panel) of 2 mononucleotide loci (*BAT25*, *BAT26*) and 3 dinucleotide loci (*D2S123*, *D5S346*, *D17S250*) exhibits the optimal consistency with IHC results concerning the MSI status detection of CRC in Chinese population, which may probably be the most suitable loci combination for MSI detection in Chinese population. In addition, there is no intratumoral heterogeneity in MSI‐H, which may be the reason for good response of MSI‐H tumors to immune checkpoint inhibitors. Based on our results, we suggest that NCI MSI panel should be applied and repeated detections or multi‐point biopsies can be spared referring to MSI detection. Larger researches are needed to define the optimal MSI detection loci as well as to clarify the dynamic changes in MSI status during tumor treatment.

## CONFLICT OF INTEREST

All authors declare no financial competing interests.
