# Supplementary figures and images for: Screening of MSI detection loci and their heterogeneity in East Asian colorectal cancer patients
Source: Cancer Med. 2019 Apr 3;8(5):2157–66. doi: 10.1002/cam4.2111 (PMC6536949; doi:10.1002/cam4.2111)

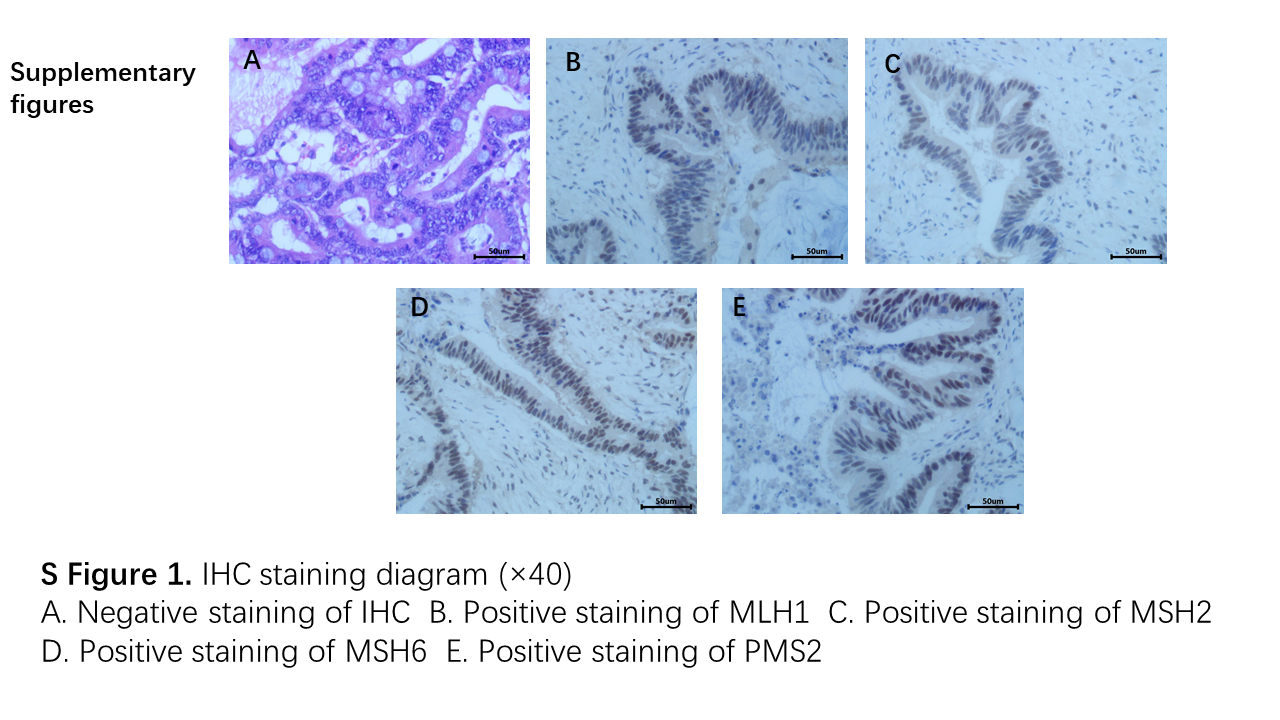

Supplement: Supplementary file 1 [file CAM4-8-2157-s001.TIF]

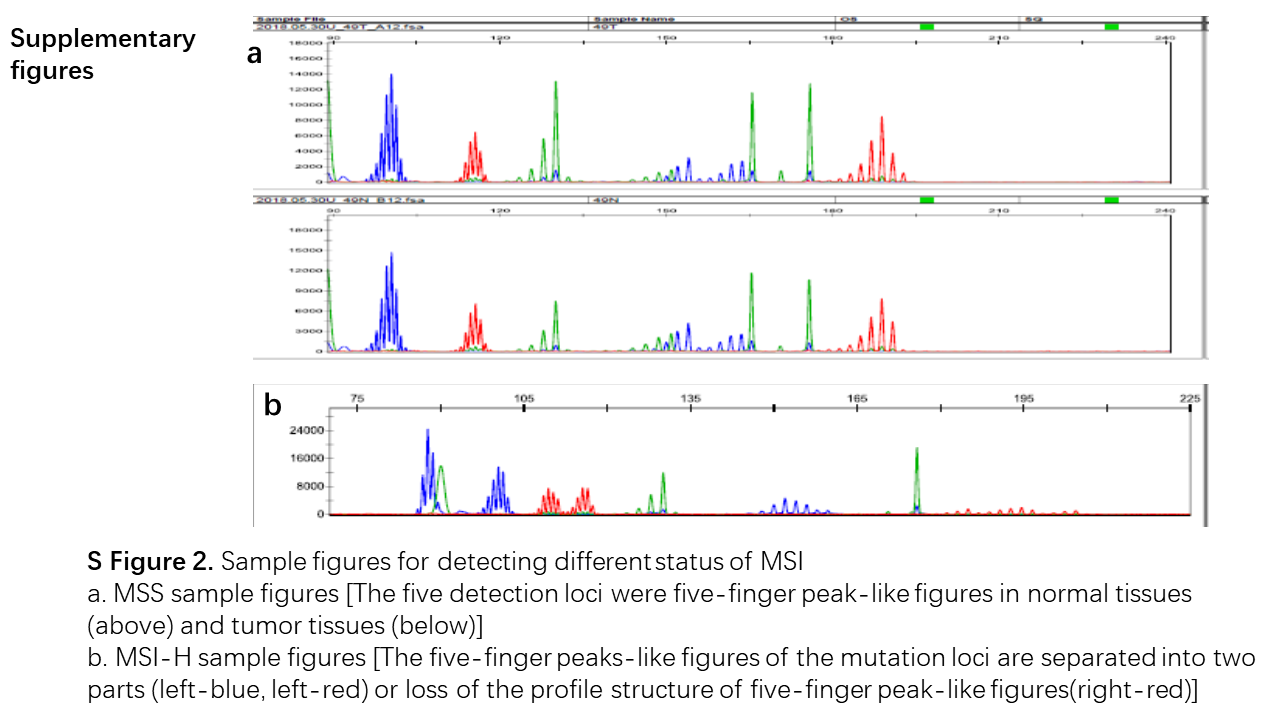

Supplement: Supplementary file 2 [file CAM4-8-2157-s002.TIF]
