# Supplementary material for: Screening of MSI detection loci and their heterogeneity in East Asian colorectal cancer patients
Source: Cancer Med. 2019 Apr 3;8(5):2157–66. doi: 10.1002/cam4.2111 (PMC6536949; doi:10.1002/cam4.2111)
Supplement: Supplementary file 3 [file CAM4-8-2157-s003.docx]

Supplementary table

**S Table 1.** Location, primer sequence and product length of detection loci

| Locus name | Chromosomal location | Primer sequence (5’ to 3’) | Size/bp |
| --- | --- | --- | --- |
| BAT-25 | 4q12 | GCT TTC CTC GCC TCC AAG AA  ACT ATG GCT CTA AAA TGC TCT GTT C | 120 |
| BAT-26 | 2p | CAG AGC CCT TAA CCT TTT TCA G  GCT TCT TCA GTA TAT GTC AAT G | 107 |
| D2S123 | 2p16 | GGC CAG AGA AAT TAG ACA CAG TGA T  TAG CCT TAG GAG CTA TTT TGA ATT G | 190 |
| D5S346 | 5q21/22 | TCA GGG AAT TGA GAG TTA CAG GT  TGG CAT ATG AAT ACC AGG ATA GCT | 147 |
| D17S250 | 17q11.2-q12 | AAG GAA GAA TCA AAT AGA CAA TAA  TCA GCT GGC CAT ATA TAT ATT TAA AC | 157 |
| BAT-40 | 1p13.1 | ATT AAC TTC CTA CAC CAC AAC  GTA GAG CAA GAC CAC CTT G | 80-100 |
| 52H10 | NA | CCC TAA CTG TCT CTA TAA AAG A  CCC AAT CTA TCT AAC ACA TTG T | 90 |
| 50C10 | NA | CCA AAG GTT ATG CCG AGG T  CGT TCA TGC GTC TGG GCT T | 80-100 |
| D1 | 11q13.3 | GCT GCT ATT GGA GGA TCA GT  GGC TAA GTG AAG CAT GAG GT | 80-100 |
| Mfd41（D17S261） | 17p12-11.1 | CAG GTT CTG TCA TAG GAC TA  TTC TGG AAA CCT ACT CCT GA | 157-171 |
| AFM249xbla（D13S175） | 13q11 | TAT TGG ATA CTT GAA TCT GCT G  TGC ATC ACC TCA CAT AGG TTA | 101-113 |
| AFM183yc3（D3S1283） | 3p24.2/22 | GGC AGT ACC ACC TGT AGA AAT G  GAG TAA CAG AGG CAT CGT GTA TTC | 150-160 |
| Mfd26CA（D18S34) | 18q12 | CAG AAA ATT CTC TCT GGC TA  CTC ATG TTC CTG GCA AGA AT | 103-119 |
| Mfd28CA（D10S89) | 10pter | AAC ACT AGT GAC ATT ATT TTC  AGC TAG GCC TGA AGG CTT CT | 142-156 |
| AFM119xh12a（D10S197） | 10qter | ACC ACT GCA CTT CAG GTG AC  GTG ATA CTG TCC TCA GGT CTC C | 161-173 |
| AFM218xela（D11S1318） | 11p15.5 | CCC GTA TGG CAA CAG G  TGT GCA TGT NCA TGA GTG | 130 |
| AFM08lza5（D11S904） | 11p14/13 | ATG ACA AGC AAT CCT TGA GC  CTG TGT TAT ATC CCT AAA GTG GTG A | 185-201 |
| AFM248yf1（D18S69） | 18q21 | CTC TTT CTC TGA CTC TGA CC  GAC TTT CTA AGT TCT TGC CAG | 110 |
| AFM.164xe31a（D9S171） | 9q21 | AGC TAA GTG AAC CTC ATC TCT GTC T  ACC CTA GCA CTG ATG GTA TAG TCT | 159-177 |
| AFM164xe31a（D18S58） | 18q22.3 | GCT CCC GGC TGG TTT T  GCA GGA AAT CGC AGG AAC TT | 144-160 |
| TP53.PCR15.1 | 17p13.1 | AGG GAT ACT ATT CAG CCC GAG GTG  ACT GCC ACT CCT TGC CCC ATT C | 103-135 |
